# Evaluation of tractor performance, efficiency and fuel consumption in vineyard activities

**DOI:** 10.1038/s41598-025-93526-z

**Published:** 2025-03-11

**Authors:** Riccardo Alemanno, Pierluigi Rossi, Danilo Monarca, Andrea Bencini

**Affiliations:** 1 Department Of Agriculture And Forest Sciences (DAFNE), University Of Tuscia, Viterbo, 01100 Italy; 2 Marchesi Antinori S.p.A, Florence, Italy

**Keywords:** Agriculture, Tractor, Idling, Fuel consumption, LCA, Environmental impact, Fossil fuels

## Abstract

Performance analyses of mechanised vineyard activities require a reliable source of information that allows proper sizing of the tractor fleet according to field requirements and an assessment of the operating costs generated by them. To achieve that, however, a large amount of data regarding the power required by machinery and their field capacity, together with fuel consumption per hour and per hectare, is needed. This research, based on CAN-bus raw data collected from narrow tractors of 75 kW through a farming management information system (FMIS) system and then processed through a Python package called Vineyardutils created by the authors resulted in a dataset that summarises 374 labelled mechanised operations over a total of 717 working hours. The summary of each operation specifies its type, duration, idle time, speed and engine parameters such as temperature, engine speed, and torque; in addition, the dataset also includes the terrain slope, fuel consumption and size of the working area. As a result, the lowest values of fuel consumption per hour and per area have been estimated for activities such as fertilisation (4.95 ± 1.20 l/h, 3.73 ± 1.48 l/ha), whereas the highest values belong to harvest operations (14.70 ± 3.39 l/h, 11.69 ± 4.68 l/ha); regarding field capacity, values range from 0.54 ha/h for leaf removal to 4.46 ha/h for multirow crop protection. Furthermore, correlations have been found regarding environmental temperature and requested power, with evidence for most of the activities. Future developments can include different tractor engine setups and additional field data.

## Introduction

The identification of the optimal working parameters and requirements of agricultural machinery, given their different impacts on investments and operating costs, is a key element in the sizing of farming fleets and in determining a reduction in negative effects on the environment^1^, which is observable in the assessment of the carbon footprint of vineyard management. The process of machinery optimisation must also consider the requirements of each activity type and their weights on the total operating time, plus a fair degree of flexibility to overcome machinery downtime: such consideration can often lead to an excessive increase in the number of available machines, hence generating higher and unwanted investments.

Research has focused on assessing emissions in viticulture as part of life cycle assessment processes, with the purpose of estimating carbon dioxide emissions per functional unit. In this context, previous works have calculated average emissions per bottle^2^; however, they have used overall consumption data and have not focused on the impact of each agricultural task. With respect to these specific aspects, such as fuel consumption per work area and necessary power, some research has been performed for crops^3^, but not enough data have been collected from vineyards. Attempts to create predictors for tractor fuel consumption have been made^4^; however, the variability in field operations and different engine setups results in complex or unfeasible applications given the lack of test data on narrow tractors and on vineyard mechanised activities.

Valuable sources of information are represented by the data generated by agricultural machinery and by field or environmental data, which can highlight patterns that strongly affect operating costs or that might indicate if the selected machinery is fit for business purposes. In vineyard management, such data are widely available, and operation logbooks are also highly recommended and sometimes even mandatory for regulatory compliance regarding the use of pesticides or food traceability, certifications and sustainability practices. An assessment of the productivity of the overall invested energy, including fertiliser, electricity and power consumption of buildings, can also be performed^5^. The presence, or in other cases, the need to fill an operation logbook guarantees the possibility of matching the data with machinery information collected from farming management information systems (FMIS): this possibility allows the tracking of field-specific operating costs, such as fuel consumption and idling^6^.

One of the remaining limitations in the use of agricultural big data is certainly the limited interpretation of raw data, the usability, the narrow bandwidth in rural areas, and the lack of data calibration^7^. The management of large volumes of data by not yet qualified and not yet specialised employees also represents a social challenge for the implementation of agricultural Big Data^8^.

Although operating costs have been largely defined, methods to detect working cycles have been created^9^, and bench analyses have been performed regarding tractor efficiency^10^; however, detailed data on costs per hour or per area in vineyards’ operations are still lacking, and they have been calculated for few specific activities or for general applications^11^. In addition, apart from assessing the effects of engine and load transients on greenhouse gases (GHG) emissions^12^, the effects of climate change have not yet been assessed in terms of variations in the power or fuel required to carry out tasks; however, the data used to perform such analyses are still not completely reliable and may lead to overestimated outcomes^13^. Correlations between power take-off (PTO) usage and fuel consumption have been identified for general agricultural purposes^14^, but they do not provide specific information regarding correlations with torque, engine speed or engine coolant temperature, which is related to environmental temperatures; additionally, some vineyard operations are performed at specific PTO speeds or even without using PTO at all, so further analyses are necessary.

Finally, important information could be drawn from a study on engine parameters regarding the effects of environmental temperatures on field performance: it is expected, in fact, to find correlations between engine coolant temperatures and environmental temperatures, and the same is true for coolant temperatures that influence oil engine temperatures, which, in turn, affect engine performance and the produced torque. Soil characteristics such as texture, or implement types, might also influence tractor metrics. Given that engine speed is often kept constant during field operations to maintain a constant PTO speed, any variation in torque generates a variation in power: although these relationships are well known, no studies have been conducted on this subject.

Given this context, the authors aimed to fill the gap in tractor performance data and therefore focused on estimating tractor fuel consumption per hour, machinery field capacity and fuel consumption per hectare for every activity that is carried out in a vineyard; in addition, the authors wanted to explore the correlation among environmental temperature and requested power to define its effect, potentially resulting in differences in fuel consumption.

## Methods

Tests were performed in 26 different vineyard fields in Tuscany, Italy. Field areas, which had an average width/length ratio of 0.83, ranged from a minimum of 2 hectares to a maximum of 8 hectares, while most of them were larger than 5 hectares. Monitored activities took place over a period of a full year, starting from May 1st, 2021, to the end of April 2022, with the aim of covering all the activity types that take place in a vineyard. The research was based on data collected from tractors while carrying out activities, which were also manually logged by farm management for compliance purposes. Operations were carried out, and therefore monitored, only during acceptable weather conditions to avoid hazards and to reduce drift effect caused by wind or procedural issues related to rain. To properly handle data and compare similar tasks, the activities have been classified as prepruning, shoot thinning, shoot removing, leaf removing, soil tillage, sward & soil tillage, light ridging, ridging, unridging, mulching, fertilisation, crop protection (both single- and multirow operations) and harvesting.

The raw data were consequently processed, and a dataset was created for each activity to provide statistics and obtain useful information regarding possible correlations among the data. A description of agricultural machinery, data acquisition and processing is provided in the following paragraphs.

The research involved 6 identical narrow tractors with a power of 75 kW and 408 Nm of maximum torque, which is a common engine power used in the context and that range from 55 to 90 kW. All the tractors, which had operational lives of approximately 3500 h, were equipped with GNSS systems and were used for all the activities that were carried out in the vineyard. Different operating machines, i.e., equipment that can be connected to tractors, were used to carry out activities that can be considered common vineyard operations and particular tasks for extraordinary maintenance. The main mechanical activities in vineyards can be divided into four categories as follows:

- canopy management;
- soil management;
- Crop protection;
- harvesting.

A description of the operating machines is shown in Table 1.

**Table 1:** Description of operating machines.

Type of activity	Type of operating machine	PTO Usage	Number of Rows
Prepruning	Disk prepruning machine	Yes	1
Shoot thinning	Trimming machine (with rotary blades)	Yes	1
Shoot removing	Sucker removal machine	Yes	1
Leaf removing	Leaf remover machine from the canopy	Yes	1
Soil Tillage	Disc harrow with cage roller	No	1
Sward & soil tillage	Chisel with 5 anchors and star roller	No	1
Light ridging	Double disc ridger	No	1
Ridging	Dual interrow tool carrier with dual hydraulic disc ridger and contour follower. Powered by the fluid drive system of the tractor	No	1
Unridging	Dual interrow tool carrier with dual hydraulic disc ridger and contour follower. Independent fluid drive system	Yes	1
Mulching	Mulcher for the control of grass growth and plant residues	Yes	1
Fertilisation	Centrifugal Fertiliser Spreader	Yes	1–2
Crop protection	Trailed straddle atomiser (multirow, MR)	Yes	2
Crop protection	Trailed straddle nebuliser (single row, SR)	Yes	1
Harvest	Trailed straddling harvester	Yes	1

Activities mostly work on each vineyard’s rows, but fertilisation and crop protection are mostly carried out by working on two rows simultaneously. Operating machines used for vineyard management, as shown in Table 1, represent common farming equipment in a flat and/or hilly agricultural context, such as chisels, atomisers, shredders and spreaders, to facilitate future comparisons with other towed equipment. Many nomenclatures used are not univocal for these activities and machines; there are several synonyms, such as “ridging” and “earthin-up” or “hilling”.

Raw data from CAN-bus (Controller area network bus) readers were collected every minute and sent to a remote FMIS system maintained by tractors’ manufacturer. Data was transmitted through a 4G mobile internet connection by a System on Module (SoM) computer, which was able to compress data and attempt a connection to the server: in case of failure in transmission, it would have attempted transmission again later together with the next packet of data. This method has already been used to determine activity costs per hectare^15^. The list of variables that the system was able to collect is shown in Table 2.

**Table 2:** Raw data collected from tractors.

Variable	Unit	Description
Date	dd/mm/yyyy	Standard date format in day, month, year
Time	hh: mm: ss	Standard time format in hours, minutes, seconds
Latitude	decimal degrees	Standard latitude data format. Variability depends on Geometric Dilution Of Precision (GDOP).
Longitude	decimal degrees	Standard longitude data format. Limitations and variability according to GDOP as for latitude.
Engine hours	hours	Total operational time of the engine [± 0.01 h]
Ambient temperature	°C	External temperature [± 0.1 °C]
Engine coolant temperature	°C	Temperature of the engine coolant [± 0.1 °C]
Engine oil temperature	°C	Temperature of the engine oil [± 0.1 °C]
Relative engine torque	percentage with no decimals	Percentage of torque in relation to the maximum torque assessed on the dynamometer break, a peak value which corresponds to 408 Nm for the tractors covered by this study
Actual engine torque	percentage with no decimals	Percentage of torque in relation to the maximum available at every specific engine speed.
Fuel tank level	percentage with no decimals	Level of tank, where 100% was 85 l [± 0.425 l]
Engine speed	rounds per minute (rpm)	Standard format for shaft speed
PTO speed	rounds per minute (rpm)	Standard format for shaft speed
PTO state	boolean	It indicated if the PTO was powered or not
Transmission state	boolean	It indicated if transmission was on or not
Wheel speed	m/s	Standard format for vehicle speed

Variability of tractor data was limited by design, since authors avoided to include operations which might have had a tendency of generating abnormal values. The raw data from the tractors did not include elevation data. To overcome this issue, elevation data have been generated from a digital elevation model (DEM) with a resolution of 10 m^16,17^^,^ via latitude and longitude data provided by a tractor: this approach enriches the dataset with elevation, avoids the generation of abnormal slope values, and allows the possibility of validating tractor paths by evaluating the consistency of latitude and longitude points during data refinement processes The DEM also facilitates the assessment of fuel tank level, a parameter that must be collected under reasonably fair slope conditions.

**Fig. 1:**
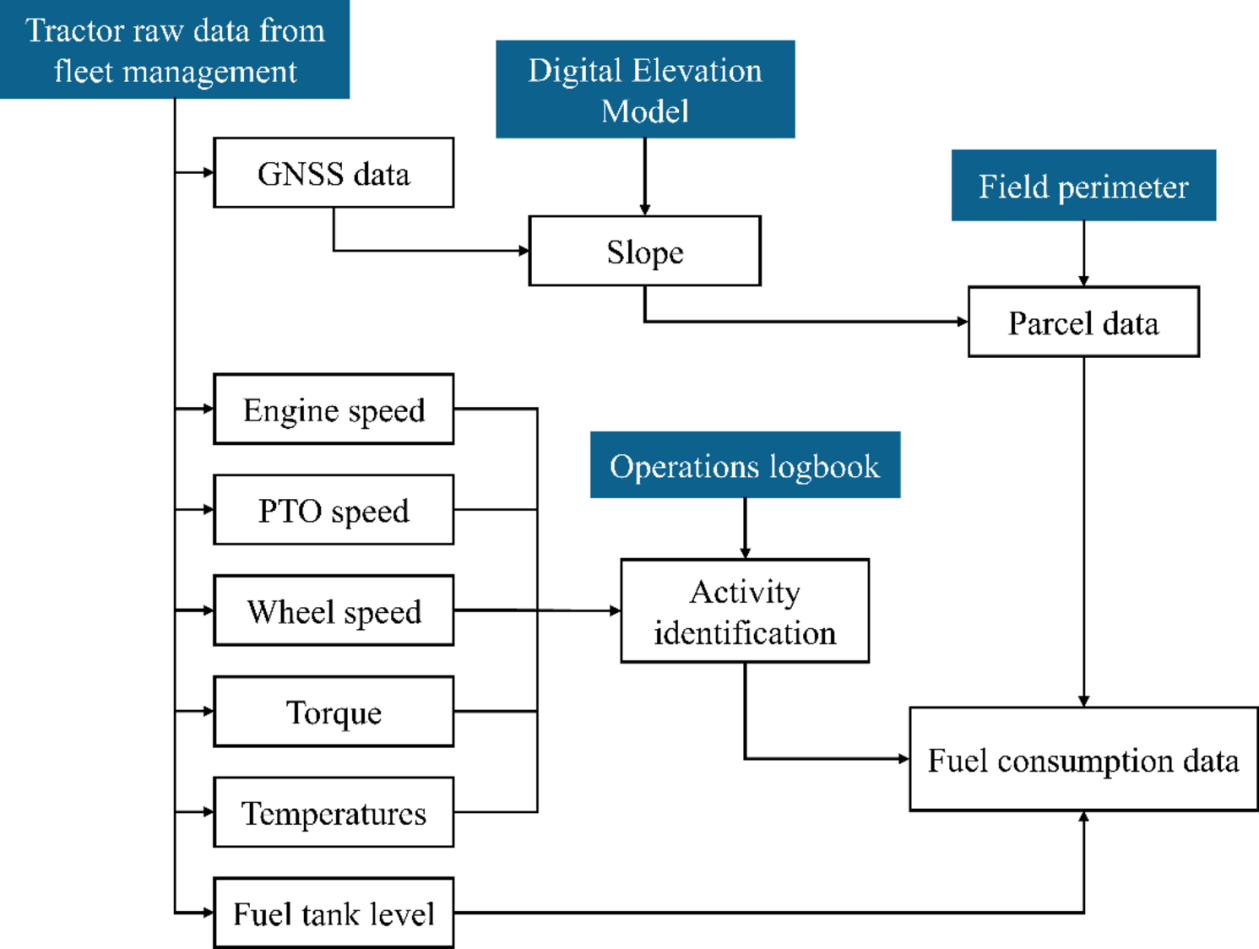
Flowchart of data processing; input data are represented in blue boxes.

As previously mentioned, in fact, a large variety of raw data have been gathered from machinery and from the environment, but further processing and integrations were necessary to generate useful information from them. The main goal of the processing was first to match the operations logbook with fleet management data in FMIS to be able to produce specific statistics on fuel consumption and work contexts for every single activity stored in the dataset; this task was fundamental to classify the activities and avoid overlapping different working conditions with similar engine parameters. In addition, the perimeter of the parcels was used to segment activities that took place in different parcels instead of storing them in a single summary entry; this reduces the quantity of data and prevents the loss of information over fields that might have had different conditions. The flow chart of raw data processing is shown in Fig. 1.

The flowchart in Fig. 1 shows how fuel consumption data can be achieved. This information comes with an error of 0.425 l given the uncertainty related to tractor tank level, but is an acceptable margin considered that monitored operations lasted more than 60 min in 75% of cases, with a minimum of 30 min for a limited number of entries. Data processing has been performed through algorithms written in Python language through a package created by the authors and published on Pypi.org as “Vineyardutils”^18^ with an open-source licence. The logic behind the algorithm follows the flowchart represented in Fig. 1, optimising the process for large datasets, providing the possibility of running it on different operating systems and achieving the desired result via the following methods:

- GNSS data (latitude, longitude) are integrated with the DEM: the algorithm binds to the tool created by the Open-Source Geospatial Foundation called OSGeo4w, which contains the geospatial data abstraction (GDAL) library that crops the DEM according to the limits of GNSS coordinates, providing faster processing. Although GDAL is available for many environments, compatibility issues are avoided in this way. The integration of DEM with GNSS data provided the possibility of performing consistency checks among the coordinates that belonged to the same row;
- Slope determination: functions related to this task provide slope calculations among geospatial coordinates in the dataset and ensure that slopes are reliable and consistent in the tractor path they belong, keeping them as reference for further data analyses only if coordinates are aligned with a maximum error of 5 degrees (forming the shape of a straight line which is typically indicating farming activities, hence angles from 175° to 185°) and if they follow the expected slope. This ensures that slope statistics are not calculated while the tractor is turning and keeping only slopes calculated inside the vineyard rows;
- Activity identification: Given that data are generated every minute, each dataset entry represents an instantiation of the operations that were being carried out. To obtain the most valuable information, functions first round the values and then calculate the modal values for each variable. Abnormal results such as full-scale values, zeros, and nulls are automatically discarded by these functions;
- Idle ratios: GNSS data and wheel speed were used to determine if a tractor was idling. An activity that resulted to be idling for more than 30 consecutive minutes was considered ended, while other data in which the tractor was not moving has been instead accounted as idle;
- Parcel data analysis and discretisation of the activities in the dataset: To provide a summary of the operations divided into single parcels, the coordinates of each dataset entry have been checked on a georeferenced map of the fields to determine if they were working within the perimeter of a parcel and adding the information regarding the parcels to which they belong to the dataset. The functions of the library check this information to determine if the tractor moved to another field or if the time that passed from one entry to another is greater than a user-defined threshold: if so, the dataset entries that have been processed are summarised as one single activity, and the area is computed on the coordinates that belong to the activity;
- Fuel consumption estimation: Based on the output of previous functions and taking the levels of the fuel tank at every entry that belongs to a single activity, an estimation of fuel consumption per hour and per area is calculated1. Notably, for these functions, the time variable is calculated according to the idle ratio to avoid the possibility that long pauses might affect the estimated consumption. On the basis of the most similar and most suitable slopes at the start and end of the activity, the initial and final slopes are presented together with fuel consumption data for further filtering and analysis in the preprocessing stage.

The information processing allowed the generation of a dataset in which a summary of each activity is present, which has been published in Mendeley Data^19^; apart from information on directly collected data, the dataset al.so contains calculated power requests, idling, average slopes, slope variations from start to finish, fuel consumption per hour and fuel consumption per hectare. The only information that has been omitted is georeferenced data for both privacy purposes and because it would have not been of interest for the research: the dataset, although it contains information regarding the size of the area covered by every activity, which has been calculated on georeferenced data. Starting from that dataset, information regarding fuel consumption statistics and correlations among variables for each activity has been produced; in addition, the produced dataset could provide material for correlation analyses, especially regarding the impact of the environmental temperature on the requested power.

Data processing also involved the removal of abnormal values in slopes, idling, fuel tank levels and activity durations. This step was necessary to avoid that site-specific field conditions or farm-specific routines generated by haste could affect the integrity of the whole dataset., Filtering of raw data provided a dataset of 374 activities over 717 h of field operations, including the average idling, speed and power required by the tractors alongside the calculated field capacities and fuel consumption rates per hour and per hectare.

## Results

The resulting dataset generated by preprocessing activities is summarised, as general overview, in Table 3 where data are grouped by activity, which also displays the number of working hours and observations that have been involved for each of them.

**Table 3:** Summary of the operational conditions for each vineyard activity.

Activity	Time (h)	*N*	Idle ratio (%)	Speed (km/h)	Torque (Nm)	Power (kW)	Fuel per hour (L/h)	Field capacity (Ha/h)	Fuel per area (L/ha)
Shoot thinning	34.10	21	12%	4.66	158.73	34.30	8.53 ± 2.82	1.22	7.68 ± 2.63
Fertilisation	15.15	10	10%	5.79	134.64	20.64	4.95 ± 1.20	1.50	3.73 ± 1.48
Leaf removing	42.20	16	19%	2.43	153.77	30.73	5.42 ± 1.52	0.54	10.30 ± 2.90
Sward and soil tillage	104.47	63	8%	4.86	200.89	38.11	7.59 ± 1.93	1.61	5.94 ± 3.36
Soil tillage	37.25	17	8%	2.57	132.48	17.12	5.26 ± 1.58	0.74	8.52 ± 3.84
Light ridging	44.58	23	20%	4.89	83.20	9.21	7.64 ± 1.97	1.72	4.82 ± 2.56
Prepruning	76.67	39	11%	3.61	180.67	35.18	8.86 ± 1.13	0.99	11.32 ± 4.29
Unridging	35.90	15	6%	3.81	187.68	35.49	6.59 ± 1.79	1.24	6.19 ± 2.88
Ridging	145.40	77	10%	2.77	171.15	33.51	8.65 ± 1.73	1.03	9.78 ± 3.66
Shoot removing	75.27	29	12%	2.78	193.45	36.68	9.87 ± 1.76	1.16	10.53 ± 4.76
Crop protection SR	32.36	19	4%	6.85	288.18	50.01	12.69 ± 2.32	2.52	5.24 ± 1.42
Crop protection MR	17.75	18	6%	7.78	349.07	61.12	13.41 ± 1.87	4.46	3.10 ± 0.763
Mulching	27.05	14	5%	5.04	194.38	36.73	8.56 ± 1.41	1.87	5.34 ± 2.35
Harvesting	23.78	10	18%	4.86	315.97	60.37	14.70 ± 3.39	1.54	11.69 ± 4.68

Activities reported in Table 3 are meant to best represent a comprehensive list of activities that are carried out in a standard vineyard conventional farming business, in order to provide specific management conditions for each of them including idling, speed, field capacity, power and overall time. Data divided by activity type shows a major impact of idling on leaf removal and harvesting, whereas its impact on the overall time of operations is reduced to 5% for crop protection and shredding; part of the idling time is indeed justified, since a large amount of time is spent in tuning towed equipment and should be considered physiological to avoid damaging the vineyard or machinery itself resulting in extra costs for repair and maintenance.

Another parameter that deserves consideration is the field capacity, which varies from the lowest value of 0,54 ha/h for leaf removing to 4,46 ha/h for multirow crop protection. In addition to having an obvious dependency on speed, this parameter is also affected by the need to work on each single row or by an increase in idling times generated by the necessity to perform adjustments and additional checks more frequently for activities that involve implements that require extra care when used. Activities that involved the use of chisel, for example, were meant to break compacted soil with vertical anchors to control grass growth and therefore took place at most 15–20 cm below soil surface, resulting in faster operations and higher field capacity if compared to pure soil tillage that, instead, involved a disc harrow which covered the whole row width at given layout limits.

The number of operations per activity stored in the dataset is strongly affected by their seasonality and by the possibility of carrying them out manually or with specific self-propelled machinery, such as in the case of harvesting and field preparation activities. In addition, the variables shown in Table 3 should not be taken as sheer numbers since they have hidden correlations that deserve to be deeply analysed. For this reason, the authors also provide additional information regarding the calculated parameters of fuel consumption per hour and per area, from which it can be possible to understand the variability of the estimations.

## Fuel consumption per hour

**Fig. 2:**
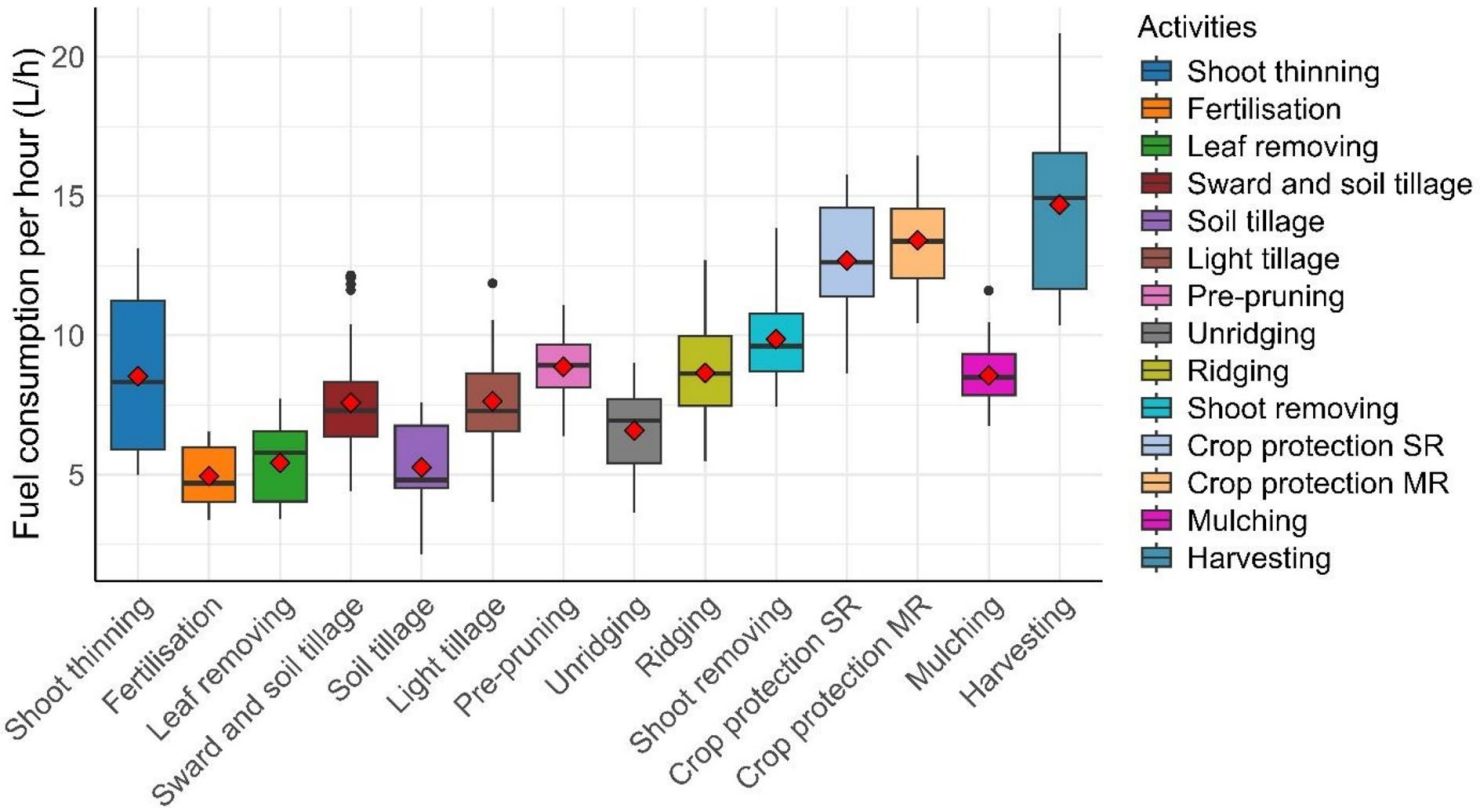
Hourly fuel consumption per activity. The red dots represent the mean values.

The data previously shown in Table 3 keep into account the standard deviation of the measurements, plus the measurement error of 0.425 l given by the instrument. A deeper analysis that highlights the details of the hourly fuel consumption measurements is presented in Fig. 2.

Given that fuel consumption is calculated according to tank levels, the error in the estimations is due to the uncertainty in the measure, which is stored as a percentage with no decimals; this aspect means that 1% point corresponds to 0.85 l, and therefore, the error can be estimated to be ± 0.425 l per hour. The activities with the highest fuel consumption resulted in harvesting and crop protection, whereas those with the lowest values were fertilisation and leaf removal. Most of the mean values were close to the median values (with some exceptions, such as harvest data), and the gap between the 2nd and 3rd quartiles was limited for many activities.

## Fuel consumption per hectare

**Fig. 3:**
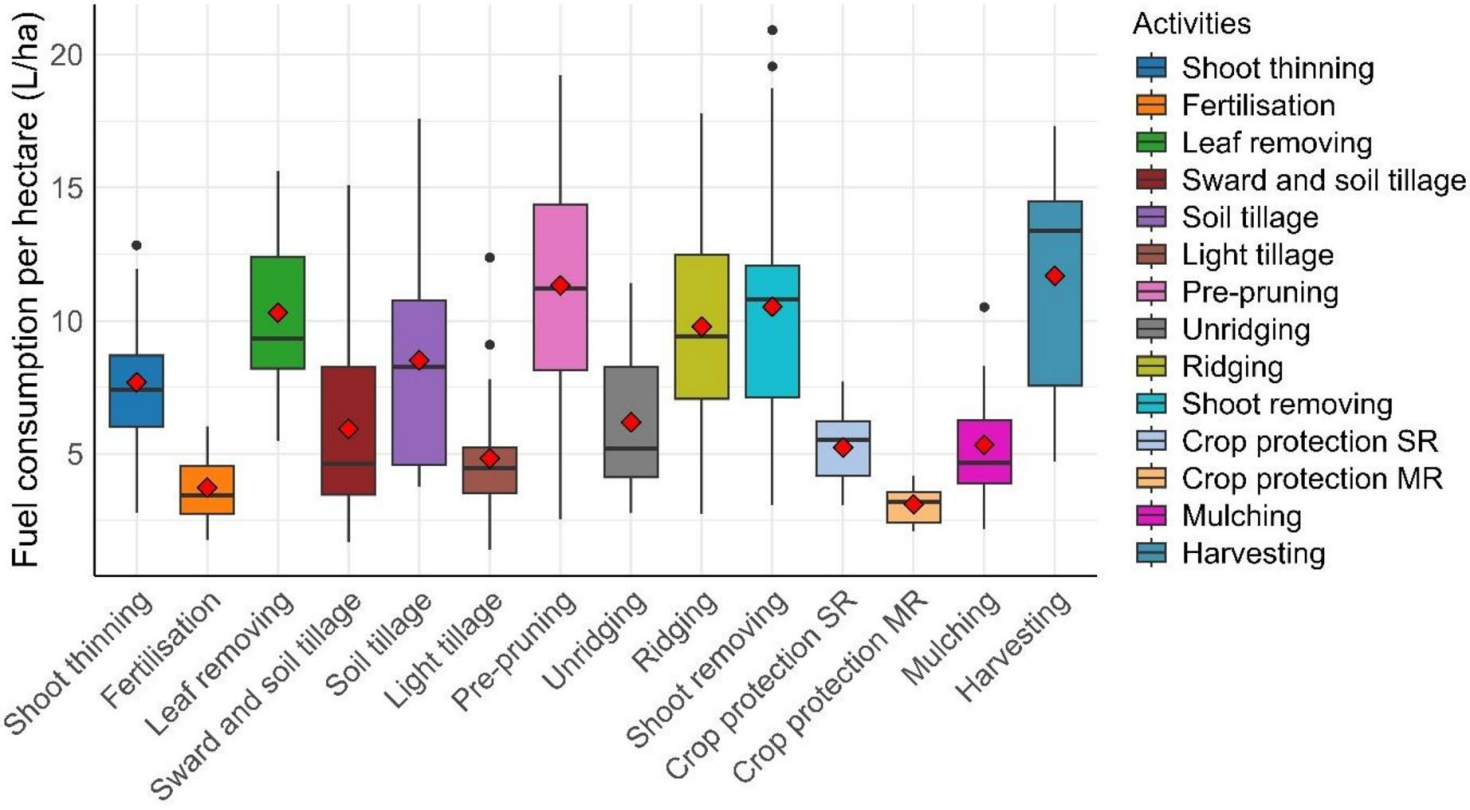
Area-based fuel consumption per activity. The red dots represent the mean values.

Further analyses involved the estimation of fuel consumption rates per worked area for each activity. In this case, variables such as speed, rows covered, and idling have a great influence on field capacity and hence also on fuel consumption, with an inverse relationship. Notably, all the fields involved in the analysis have comparable surfaces, and their shape is mostly rectangular, involving a limited number of turnings, which also reduces the chances of mispositioning the tractor by the global navigation satellite system (GNSS), which could lead to erroneous calculated trajectories. Data regarding fuel consumption per worked area, divided into each activity, are shown in Fig. 3.

As previously mentioned, the effect of idling is clearly visible for leaf removal compared with fuel consumption per hour, while outliers are generated by fields with shorter rows that require tractors to perform a higher number of turns independently of the field area. Harvest remains one of the most fuel-consuming activities, while field preparation values can vary significantly since they are non-standard operations in which several parameters present high variability. Notably, for fuel estimations per hour, most activities have ± 2 l/ha of variability from the mean values to the 2nd and 3rd quartiles, and except at harvest, all the mean values are close to the median values.

## Impact of the environmental temperature on power requests

**Fig. 4:**
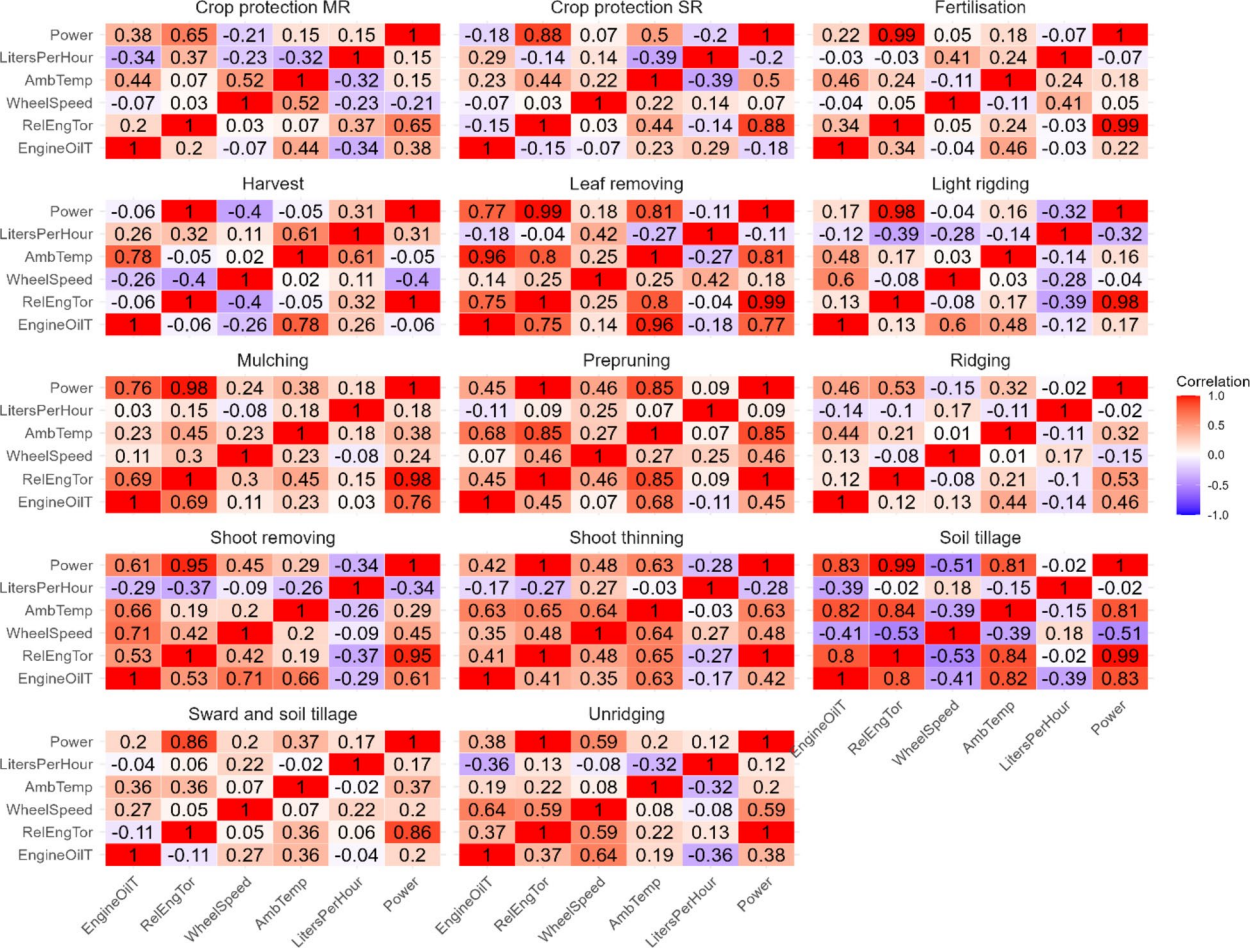
Correlation plots for each activity.

Correlations among variables, especially those that authors expected to find, had to be analysed for each single activity owing to different requirements among them. A correlation analysis is hence shown in Fig. 4.

**Fig. 5:**
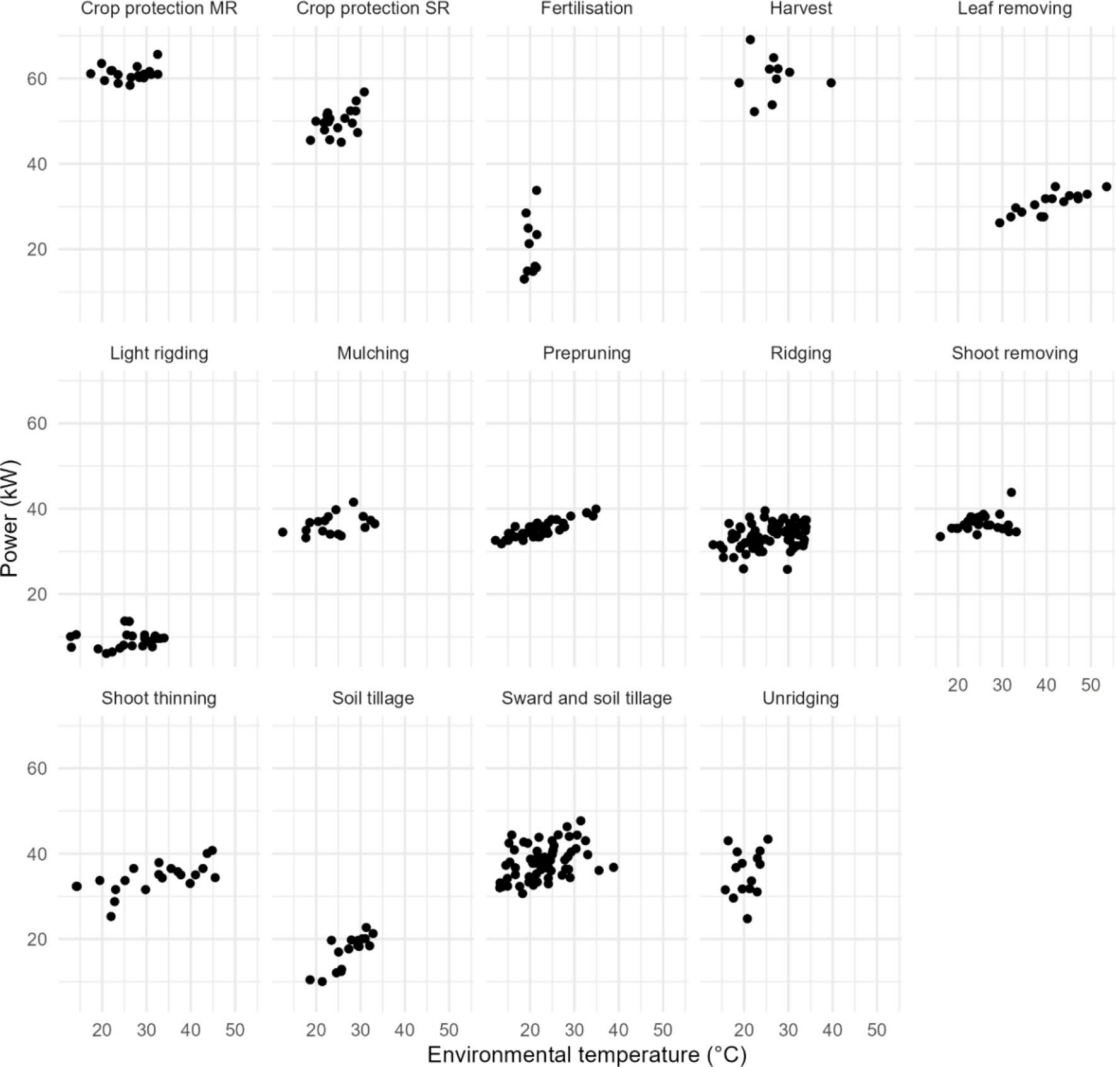
Dispersion plots of the ambient temperature versus the required power.

Correlation plots confirm the well-known impacts of the ambient temperature on the engine oil temperature, engine oil temperature on the relative engine torque for most activities, and a high correlation between the engine torque and power. The relationship between the ambient temperature and the requested power is also evident, and further information can be drawn from dispersion plots for each vineyard activity, as shown in Fig. 5.

The correlations appear to be, at first glance, linear for most activities; some of them, such as harvesting and fertilisation, are strongly affected by quantities and by the limited number of observed activities in the period of analysis and consequently correlations cannot be seen, whereas activities such as crop protection are performed by different types of towed equipment and in different working modes, which is why they have been split into two different configurations. To further analyse possible correlations among data, an analysis of the variance (ANOVA) has been carried out to detect linear regression information. ANOVA is able to provide an indication on operational patterns even with a limited number of entries, and since the aim was to verify patterns inside an imbalanced activity-based dataset the selected tool was fit for the purpose. The results of ANOVAs for each activity are shown in Table 4.

**Table 4:** Results of the ANOVA test for correlations among the environmental temperature and the required power.

Activity	Correlation	Slope	Intercept	*p* value	F-statistic
Prepruning	0.8538	0.3009	28.5370	4.889929e-12	99.5334
Leaf removing	0.8125	0.3156	17.8493	1.306397e − 04	27.2096
Soil tillage	0.8087	0.8088	-5.05244	8.493975e-05	28.3544
Shoot thinning	0.6347	0.2245	27.193	0.0019	12.8170
Crop protection SR	0.4963	0.4308	39.3591	0.0306	5.5552
Mulching	0.3809	0.1504	32.7056	0.1313	2.5451
Sward and soil tillage	0.3695	0.2547	32.2198	0.0028	9.6451
Ridging	0.3180	0.1549	29.5447	0.0048	8.4353
Shoot removing	0.2940	0.1383	33.2224	0.1216	2.5548
Unridging	0.2025	0.3798	27.7015	0.4691	0.5560
Fertilisation	0.1765	1.1297	-2.25565	0.625	0.2574
Light rigding	0.1589	0.0487	7.95418	0.4688	0.5442
Crop protection MR	0.1499	0.0553	59.6731	0.5526	0.3679
Harvest	-0.0462	-0.04	61.4310	0.8990	0.0171

Most of the vineyard activities have reported a relevant correlation (correlation values that range from 0.8538 − 0.3180 or ANOVA p values below 0.05), and the linear correlations also show very similar slopes. Power-tillage, suckering, shredding and light tillage also show a certain degree of correlation, although a reduced impact of temperature over the requested power is observed given nearly halved slope values. Activities such as vineyard harvest and fertilisation did not show correlations among temperatures and requested power, but fewer data were also available for them; last, crop protection did not show correlations among the environmental temperature and requested power.

## Discussion

The possibility of calculating tractor fuel consumption per hour and per hectare, together with precision agriculture practices and telemetry-based information, can further improve the estimation of greenhouse gas levels^20–22^. The calculation methods can be improved by increasing the data transmission rates; however, this would increase wireless network bandwidth requirements and might reduce live data availability in environments that are hard to reach for mobile broadbands such as LTE or 5G. A local data storage can overcome such issues but if limited connection conditions persist for longer times this solution might result in an unsustainable impairment between data generation rates and network’s upload speed. In addition, a generalised lack of data from towed equipment also increases the error margins, but future developments in farming machinery will likely provide a steady increase in the variety and availability of such information.

The correlations found for the environmental temperature and requested power for most of the activities represent a starting point for assessing the impact of temperature on fuel consumption and GHG emissions in mechanised vineyard activities. However, additional analyses are required to investigate if the correlation and its magnitude that have been found for narrow tractors are also present in other tractor engine configurations under different working conditions. Field data and specific engine configurations would also offer much more awareness of the variables that have an impact on the correlation, apart from wheel speed, PTO speed and implements that were all kept constant already for each single activity in this study; ; notably, engine efficiency itself is thermodynamically affected by the environmental temperature, so estimations should also consider such variability. An additional consideration that is worth mentioning is that the field operations that are mostly affected by the correlation between environmental temperature and requested power might also be carried out in different moments of the year according to seasonal variability and field latitude. Regarding idle ratios, these are an important parameter for calculating tractor operating costs and they also represent an useful benchmark to assess if farming management improvements can be done.

The methodology used in this research could be further adapted to investigate telemetry in other crops, resulting in a broader set of decision-making opportunities. Future developments could involve several aspects of the research, such as different tractor engine configurations, towed equipment with digital farming capabilities, faster rates of data harvesting, real-time kinematics (RTK) GNSS navigation systems and possibly more accurate field information, such as DEMs (digital elevation model) with higher resolutions and soil data. Combined with the capabilities of machine learning, the vast volume of data can be used to solve problems such as crop management and agricultural decision-making^23,24^. Therefore, another aspect to explore in the future is the use of a larger, multifield dataset, which could serve as the basis for supervised machine learning applications.

## Conclusion

The findings of this research provide a quantification of fuel consumption for each vineyard activity performed with narrow tractors of average power capacity. Within the previously discussed field conditions and limitations, these values ranged from a minimum of 4.95 ± 1.20 l/h (3.73 ± 1.48 l/ha) for fertilisation activities to a maximum of 14.70 ± 3.39 l/h (11.69 ± 4.68 l/ha) for harvesting. These results have also been presented alongside specific field capacities, which are influenced by multiple factors: the lowest field capacity was observed in leaf removal activities (0.54 ha/h), while the highest was observed in multirow crop protection activities (4.46 ha/h). These findings show that leveraging CAN-bus telemetry data can provide a detailed quantification of energy demands, fuel consumption and performance metrics for various vineyard mechanised activities. Accurate fuel consumption estimations can significantly enhance Life Cycle Assessment (LCA) analyses by facilitating specific evaluations of GHG emissions generated by vineyard machinery. Additionally, understanding the influence of environmental temperatures on machinery power demand can also play a key role in linking the effects of different climate conditions to variations in field performances.

The dataset produced for this research^19^, which is freely available, can serve as a resource for future research studies that investigate power requirements or idling times in agricultural machinery for economic analyses. Moreover, potential applications with additional data can extend beyond the limitations that have been previously discussed in this study: for instance, it could be used to explore the impact of field conditions such as slope and soil characteristics on machinery performance or analyse the variability of fuel consumption and field capacity for a specific vineyard operation performed with different farming equipment or tractor models. Future research in this field could also rely on the existing data processing algorithms that have been developed in this study and are available as Python package^18^, potentially enhancing their functionalities to suit specific applications.

## Data Availability

Data used for this study have been published as a dataset on Mendeley Data (doi: 10.17632/y3h5xzkjc6.1) in both comma-separated values and Microsoft Excel formats. The Vineyardutils Python library has been published at https://pypi.org/project/vineyardutils. The dataset does not contain sensitive information or personal data regarding the farming business or its workers.
